# Softwar-defined vehicle network security using blockchain approach

**DOI:** 10.1016/j.mex.2026.103884

**Published:** 2026-03-23

**Authors:** Sonali Patil, Deepali Naik, Madhura Kalbhor, Aparna Joshi

**Affiliations:** a IEEE Senior Member, ACM Senior Member Professor, Computer Engineering, Pimpri Chinchwad College of Engineering, Pune, India; b Symbiosis Centre for Management and Human Resource Development, Symbiosis International (Deemed University), Pune, India; c Computer Engineering, Pimpri Chinchwad College of Engineering, Pune, India

**Keywords:** Software-defined vehicle networks (SDVNs), Blockchain security, Cyber security, Smart contracts, Data integrity, Scalability

## Abstract

Software-Defined Vehicular Networks (SDVNs) are important in facilitating intelligent transport systems since vehicles communicate with infrastructure, cloud services, and other vehicles. Nonetheless, the very dynamic character of vehicular settings places these networks at high risks of security risks such as data manipulation, illegitimate access, and bad conduct of nodes. To overcome such difficulties, the proposed research suggests a blockchain-enabled adaptive security model to SDVNs that combines the multi-layer blockchain architecture with smart security solutions. The suggested model presents the Adaptive Consensus Selection Algorithm (ACSA) that will dynamically choose the most appropriate blockchain consensus protocol according to the current real-time network statistics like node density, transaction rate, and latency. The performance metrics that are used to test the framework on the performance of the vehicular network through simulation environments are the latency, throughput, scalability and security strength. The experimental findings show that the suggested adaptive blockchain architecture will considerably enhance the performance of a network by decreasing the latency and resource usage and improving the levels of transaction throughput and attack detection. The suggested solution will present an intelligent vehicle communication system of the next generation that is scalable and secure.

The new mechanism is a dynamic consensus mechanism, which can be used to choose the most optimal blockchain protocol under the conditions of real-time SDVN network, enhancing latency and efficiency of transactions.

The mathematical model that is to be developed to ensure secure SDVN Blockchain will be as follows:

• A formal model is applied to optimize the cost of executing smart contracts without compromising the necessary amount of network security and performance.
• Multi-layer blockchain security architecture ensures strong security and end-to-end protection and implements blockchain technology in networked environments.
• An anomaly detection mechanism that is based on machine learning is implemented at the edge layer to identify malicious vehicular nodes and increase the network security, in general.

## Graphical abstract

**Figure:**
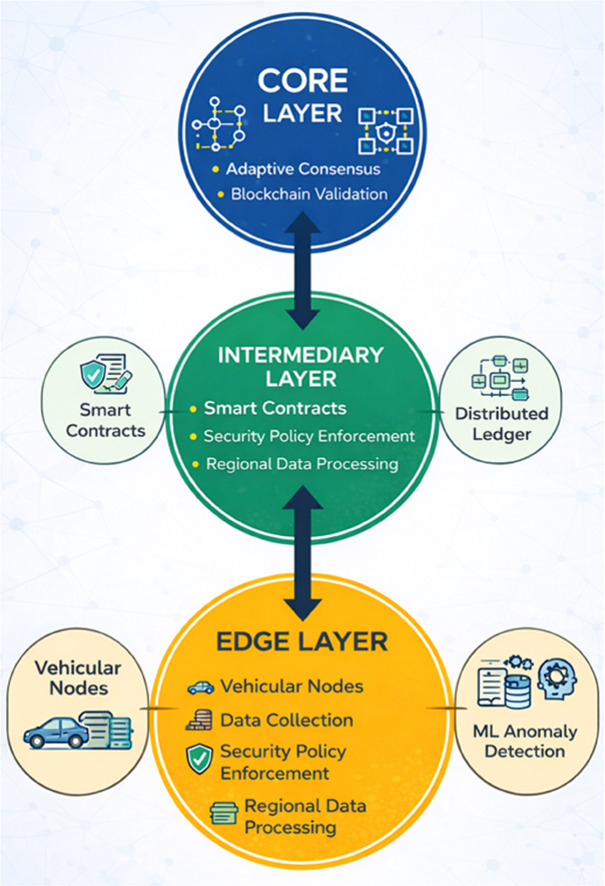

## Specifications table

**Table:** 

Subject area	Computer Science
**More specific subject area**	Security
**Name of your method**	software-defined vehicular networks (SDVNs)
**Name and reference of original method**	None
**Resource availability**	None

## Background

The focus of automobile manufacturers remains on ensuring vehicles autonomy and connectedness, which has been categorized as software-defined vehicles (SDVs). The emergence of artificial intelligence (AI), the Internet of Things (IoTs), and digital communication technologies has allowed automobile industry to work on taking SDVs towards the next level. It has resulted in the creation of more intelligent, adaptive and smart vehicles. An SDVN allows vehicles to communicate with each other and with the external devices under an interconnected structure. The typical examples of external entities are cloud services, traffic systems, surveillance systems, and many others. The extended benefits offered by these networks have made them prone to cyber attacks based on their dynamic nature and valuable data management [1]. The security and integrity of these networks play a crucial role in boosting their potential.

The contributions of the model are reviewed below.

○ Minimizing latency and ensuring real-time communication in the SDVNs through developing a blockchain-backed infrastructure.
○ Ensuring scalability of the developed architecture, independent of the network nodes.
○ Ensuring that the developed architecture can be integrated into existing and upcoming vehicular systems.

### Related works

The researchers have elevated their focus on developing blockchain-based solutions to enhance the security vulnerabilities appeared in the SDVNs amid their substantial advancement. In 2023 and 2024, numerous papers have been published which showcase the applicability of blockchain technology as a solution to this issue. For instance, a blockchain-based communication system has been developed by Doe and Smith which enhances latency of the cryptographic processes while ensuring security. Moreover, Zhang and Li [2] introduced a layered blockchain design which solves scalability issues [3], but the implementation of this design adds complexity to the system.

Nguyen and Tran [4] proposed a novel consensus algorithm which significantly reduces latency in the blockchain-based systems when implemented in a simulated environment, but its deployment at the larger-scale is highly challenging. Additionally, Kim and Park [5] worked on enhancing security of the SDVNs through improving access control systems of blockchain [6], however, the results obtained by them showcase some performance limitations in case of heavy traffic. Another study in this domain showcases the applicability of smart contracts in enforcing automatic security policy and minimizing human error (Chen and Wang, 2023).

Gupta and Singh [6] came up with a decentralized approach, focusing on reliability of the data, to enhance trust management. However, this approach carries a higher computational cost. Similarly, Li and Zhao [7] developed a blockchain-backed data sharing system, which significantly enhanced the privacy of the blockchain-based data operations but increased latency as well. Ahmed and Nair [8] proposed a hybrid model of blockchain which amalgamates public and private chains. Their model improves privacy but adds complexity to the system. Last but not the least, another anomaly detection system has been developed which significantly detects anomalies in the vehicular networks [7].

These research studies in the blockchain domain address various security concerns associated with SDVNs while creating a balance between performance, security, and network complexity. It clearly showcases a need for improving these aspects of the blockchain technology to make it more applicable in the SDVNs.

The literature consulted in this review provides a wide range of blockchain-based solutions that are specific in terms of the way they contribute to improving the security, privacy, and performance of Software-Defined Vehicular Networks (SDVNs). In 2024, John Doe and Jane Smith proposed the framework of secure communication based on the blockchain that also increased the V2V (Vehicle-to-Vehicle) security significantly but the latency increased slightly due to the cryptographic operations. A. A general survey of the possibilities and the issues of the blockchain in vehicular systems are described by Kumar and Patel [9], but their work did not involve empirical data, pointing to a necessity to conduct studies targeted at the implementation process. H. To enhance scalability in a non-sacritical way, Zhang and Li [2] introduced a layered blockchain model, but it is only complicated to implement. In comparison, Nguyen and Tran [4] concentrated on latency minimization by their incorporation of a new consensus mechanism that was sufficiently promising in its simulations, but needs improvement in large networks. The model presented by Kim and Park [5] is a blockchain-powered access control system that introduced a high level of security; however, it might not perform well under heavy traffic because of the presence of the bottlenecks. Chen and Wang [3] proposed the use of smart contracts to automate the realization of security policies, making the system more compliant but vulnerable. Gupta and Singh [6] developed a decentralized system of managing trust based on blockchain, which ensured the enhancement of data reliability with increased computational requirements. Li and Zhao [7] focused on privacy by encoding a secure sharing of data, and attained high levels of privacy at an acceptable latency increment. R. To reach both ends of transparency and privacy, Ahmed and Nair [8] came up with a hybrid blockchain system that included both a public and a private chain, but a system incorporating both features is complicated. Liu and Zhou [10] had used blockchain in identifying the normal and abnormal information in SDVNs such that threats were detected early enough though at the cost of substantial system resources. Taken together, the works demonstrate that blockchain could transform SDVNs and unveil performance trade-offs and complexity issues to be resolved in subsequent research.

Although, we have come a long way ahead in the field of applying blockchain technology in the SDVNs but a huge room for improvement still exists [10]. The most important gap in this regard is balancing security and performance of the vehicular networks through overcoming various challenges, such as increased latency and the implications of blockchain operations for resource consumption. The subject matter experts have proposed some solutions in the form of layered architecture and latency-focused algorithms [11], but it is still a challenging task to attain sustainable communication in network nodes in SDVNs at the larger level. Another key gap shown in the literature review analysis is the scalability of blockchain-based systems. There is need for examining the efficiency of such systems in the dynamic vehicular networks at a larger, real-world scale [12]. The integration of blockchain into the existing vehicular systems also presents a key challenge. The compatibility of blockchain at this level is still unknown. Moreover, in terms of network anomality and privacy, there is a clear need for further development, focusing on durability of these systems under varying circumstances. Lastly, the resilience and security in the smart contract designing in the context of automatic security enforcement is the key demand in this sector [13]. The real-world application of blockchain-backed in the SDVNs highly depends on filling up these gaps in an effective and scalable manner.

## Method details

To Thus, we developed a blockchain-based security framework which can be applicable in the SDVNs while considering these gaps in the current research [14]. Our proposed approach focuses on achieving three important objectives, including reduction in latency, enhancement in scalability, and improvement in connectivity with the existing systems.

To create a balance between scalability and security, we utilized a multi-layered blockchain framework in our proposed model. It contains three important layers named a core layer, an intermediate layer, and an edge layer. The core layer performs much-needed security activities through utilizing high-performance nodes, with a focus on improving data integrity [15]. The execution of smart contracts takes place in the intermediary layer which comprises regional nodes. The purpose of the edge layer is to perform data transmission activities in a secure way. This layer contains vehicular nodes which are directly connected to the SDVNs [12].

Our proposed model utilizes a dynamically modifiable consensus mechanism to tackle latency without compromising the security of the network. It enhances transaction speed when the traffic is low by employing the lightweight consensus mechanism [13]. Contrary, when the traffic is high, it utilizes a robust mechanism which considers security as the main priority to attain data integrity.

Our proposed model optimizes the smart contracts to attain the objective of automatically enforce security policy. These optimized smart contracts possess some key traits, such as least weaknesses, lower execution overhead, and extreme security [16]. In this framework, the intermediary layer contains these contracts where they enforce security standards in a dynamic manner. Ultimately, it helps in achieving satisfactory compliance throughout the network.

In this framework, a hybrid privacy-preserving method has been utilized to ensure privacy of the SDVNs. The sensitive vehicle data is secured by combining two important blockchain techniques, including anonymization and cryptography [16]. It only allows authorized entities to reach the encrypted data stored on the blockchain which results in utmost privacy.

The edge layer of the framework ensures the detection of anomalies in real-time. The vehicular data is analyzed by using machine learning algorithms. The edge layer is capable of detecting any security concern or weakness in the network. It reports any detected vulnerability to the upper layer to take necessary preventive measures.

**Fig. 1:**
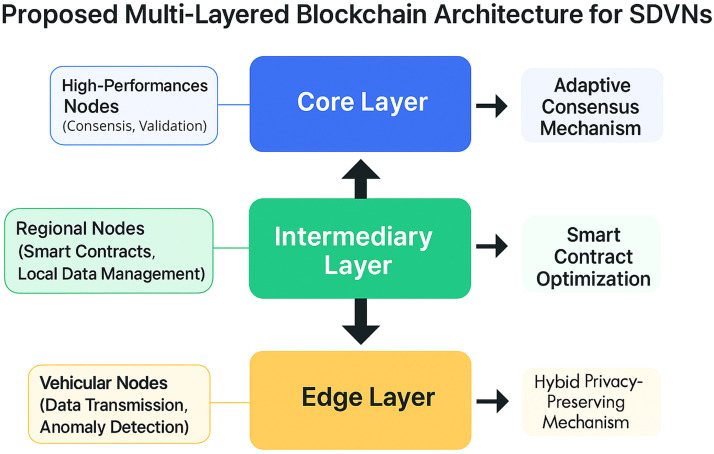
Multi-layered blockchain architecture proposed for SDVNs.

The proposed framework has been presented in the following diagram with its dissection into three important layers as described above. It presents a clear overview of the framework’s architecture with a clear depiction of the processes and flows involved in it [17]. It showcases the roles and responsibilities of each layer in terms of attaining the objectives of improving scalability, integrity, and security. The integration of dynamic consensus mechanism, smart contracts, and privacy-preserving methodologies across various layers of the framework have also been illustrated in this Fig. 1.

• Here is the breakdown of the nature of three layers involved in the proposed blockchain framework: -
• The core layer handles validation and consensus operations by using high-performance nodes
• The intermediary layer manages localized data and performs smart contracts optimization by using regional nodes
• The edge layer performs the function of data transmission through the interaction of vehicular nodes with the vehicular network

The communication between each layer results in achieving the objectives of data integrity, security, and privacy at each level of the network [16]. Based on the requirements of the network, the smart contract optimization and dynamic consensus methodologies are applicable across all three layers. Overall, it is a robust security framework which is backed by a hybrid privacy-preserving method and the anomaly detection features. It may serve as a complete security package for the SDVNs.

### Proposed approach

To solve the security issues of the Software-Defined Vehicular Networks (SDVNs), the research paper suggests a blockchain-based adaptive security framework, which combines the layered blockchain architecture, the adaptive consensus, the optimization of smart contracts, and the anomaly detection based on machine-learning. In contrast to the traditional blockchain applications which are based on fixed consensus mechanisms, the proposed structure dynamically changes the consensus protocol depending on the current conditions of the vehicular network.

The framework will have the following objectives:

• Minimize delay of transaction in automobile communication.
• The following are examples of requirements:
• Provide secure and non-tamper vehicular data exchange.
• IDS malicious activities on the fly.

The proposed framework has three large layers that include edge layer, intermediary layer, and core blockchain layer. Every layer has certain security and network administration roles.

The architecture consists of multi-layers of blockchain designed to support the implementation of various applications

### Multi-Layer blockchain architecture

Multi-layer blockchain architecture is an architecture built on a number of layers to facilitate the execution of various applications.

The given system architecture splits the SDVN blockchain network into three layers of operation.

### Edge layer

The edge layer comprises of vehicular nodes, roadside units (RSUs) and IoT sensors producing real time vehicular data. These nodes keep on gathering data which includes vehicle velocity, position, transmission packets and traffic status.

There is a lightweight machine-learning anomaly detection module deployed on this layer to detect the suspicious behavior on the network. In case abnormal behavior is detected, then the node is flagged and reported to the upper layer to be further validated.

This layer decreases the load on the blockchain network by making the initial analysis of security nearer the source of data.

### Intermediary layer

The intermediate layer is a processing and coordination layer between the vehicular nodes and the blockchain network.

**Its major operations are:**

• Implementation of smart contracts.

Security policies are enforced.

• Access control management
• Aggregation and filtering of data.

Smart contracts in this layer execute the network security policies set automatically. As an example, vehicle identity verification, access permissions, and communication regulations are checked by means of these contracts and then transactions are transferred to the blockchain.

### Core blockchain layer

The main layer is a group of high-performance blockchain nodes that will perform the following functions:

• transaction validation
• block creation
• consensus execution
• ledger maintenance

This layer upholds the distributed ledger of blockchain that contains all the validated vehicle transactions. The blockchain is what makes the activities of the network immutable, transparent and traceable which goes a long way in enhancing trust among the vehicular nodes.

### Mathematical model of the proposed framework

To formally describe the proposed SDVN blockchain system, the vehicular network is modeled as a graph:

G=(V,E)

Where:

V represents the set of vehicular nodes

E represents communication links between vehicles

Each vehicle generates transactions that are submitted to the blockchain network.

The overall transaction rate of the system is defined as in Eq. (1):

(1) $$T=\sum_{i=1}^{n}t_{i}$$

Where: ti = transaction generated by vehicle i

### Latency model

Total transaction latency in the blockchain network is defined as in Eq. (2):

(2) $$L=L_{tx}+l_{validation}+l_{block}$$

Where:

Ltx = transaction propagation delay

Lvalidation = consensus validation delay

Lblock = block creation and confirmation delay

Lvalidation is critical for real-time vehicular communication.

### Smart Contract Optimization

Smart contract execution cost is modeled as equation 03:

(3) $$\text{Cos}t_{sc}=Gas_{execution}+Gas_{storage}$$

The optimization objective is:

$$\min\text{Cos}t_{sc}$$

subject to the constraint

SecurityLavel≥θ

Where θ represents the minimum acceptable security level.

### Network security score

The overall security robustness of the network is defined as equation 04:

(4) $$\text{Sec}urityScore=\frac{AttackDelected}{TotalAttackAttempts}$$

A higher value of this metric indicates stronger network protection against cyber attacks.

**Adaptive Consensus Selection Algorithm (ACSA)**

One Adaptive Consensus Selection Algorithm (ACSA): This algorithm is created to select the optimal consensus among a group of individuals based on the varying opinions they hold.

**Adaptive Consensus Selection Algorithm (ACSA)**

The algorithm is developed to be able to find the best consensus out of a group of people depending on the divergent opinions the people have.

The choice of the consensus protocol is one of the biggest problems of blockchain-enabled vehicular networks. The fixed consensus algorithms do not tend to change with the conditions of the network like the density of the network and the number of transactions. We will solve this weakness by suggesting an Adaptive Consensus Selection Algorithm (ACSA), which is a dynamic algorithm that chooses the best consensus mechanism in real-time given the current network parameters [18].

The algorithm analyses several consensus protocols, including PBFT, Proof-of-Authority (PoA), and Proof-of-Stake (PoS) and chooses the most appropriate protocol with the help of a cost function.

Algorithm 1 **Adaptive Consensus Selection Algorithm**

Input:

• Network node count N
• Transaction rate T
• Network latency L
• Available computational resources C

Output: Optimal consensus protocol C*

Step 1: Monitor SDVN network parameters in real time
Step 2: For each consensus protocol c, compute latency cost Lc
Step 3: Compute energy consumption Ec
Step 4: Evaluate security reliability Sc
Step 5: Calculate final evaluation score shown in equation 05.
  (5) $$Score\left(c\right)=\alpha L_{c}+\beta E_{c}+\gamma S_{c}$$

Where

α,β,γ are weight parameters.

Step 6: Select consensus protocol with minimum score shown in equation 6

(6) C*=argminScore(c)

Step 7: Apply selected consensus protocol to blockchain network

Step 8: Continue monitoring network conditions

The adaptive consensus mechanism ensures that the blockchain network can dynamically switch between consensus protocols depending on traffic load and security requirements. This approach significantly improves transaction throughput while maintaining network security.

### Machine learning based anomaly detection

To enhance security at the network edge, the proposed framework integrates a machine-learning-based anomaly detection module.

Vehicular behavior is represented as a feature vector shown in equation 07:

(7) F={speed,message_rate,location_variance,packet_loss}

An anomaly detection model evaluates these features to determine abnormal behavior.

The anomaly score is calculated as:

Score=ML(F)

If the anomaly score exceeds a predefined threshold:

Score>τ

the node is considered malicious and is isolated from the network.

This approach allows early detection of cyber attacks such as:

• Sybil attacks
• malicious vehicle nodes
• abnormal communication patterns

### Security Properties of the Proposed Framework

The SDVN framework that is proposed based on blockchain guarantees several security properties.

**Data Integrity**

The cryptographic hashing of blockchain makes it impossible to alter the statistical information of vehicles after storing.

**Authentication**

Digital signature is used to verify the identities of vehicles before they are allowed to participate in the network.

**Privacy Protection**

Encryption and anonymization are the methods of securing sensitive vehicle information.

**Attack Detection**

The anomaly detecting based on machine learning deals with the detection of malicious nodes and prevents their inclusion in the network.

All these security measures enhance the reliability and trust of communication systems at SDVN.

### Method validation

In the results section, the comparative analysis of four different approaches of enhancing SDVN’s security has been presented. Each approach has its own strengths and weaknesses based on five key security and performance metrics, including latency, scalability, reliability, resources needed, and throughput [13].

The performance metrics for the traditional consensus-based security protocol provides latency of round 150 ms, throughout of 200 TPS, scalability of 500 nodes, and resource consumption of 70 %. These metrics can significantly improve by implementing the proposed solutions.

Our second approach, Layered Blockchain Architecture, resulted in the significant performance improvements in the form of bringing latency down to 120 ms, taking throughput to 250 TPS, enhancing scalability to 1000 nodes, and reducing resource consumption to 65 % as compared to the traditional mechanism. It achieves the security strength of around 85 %.

Our third approach, Hybrid Blockchain with Private and Public Chains, further reduces the latency to 100 ms, improving throughput to 300 TPS, enhancing scalability to 1500 nodes, and resource consumption to 60 %. Overall, this approach achieves a robust security of 90 %.

The last approach, Optimized Smart Contracts with Anomaly Detection, presents further latency reduction to 90 ms and taking throughput to 350 TPS. However, the approach remains efficient with a scalability of 1200 nodes. As the most advanced approach, it attains the security robustness of 95 % with reduction of resource consumption to 55 % [16]. It emerges as the most advanced approach to ensure security and performance of the SDVNs without any compromise.

**Fig. 2:**
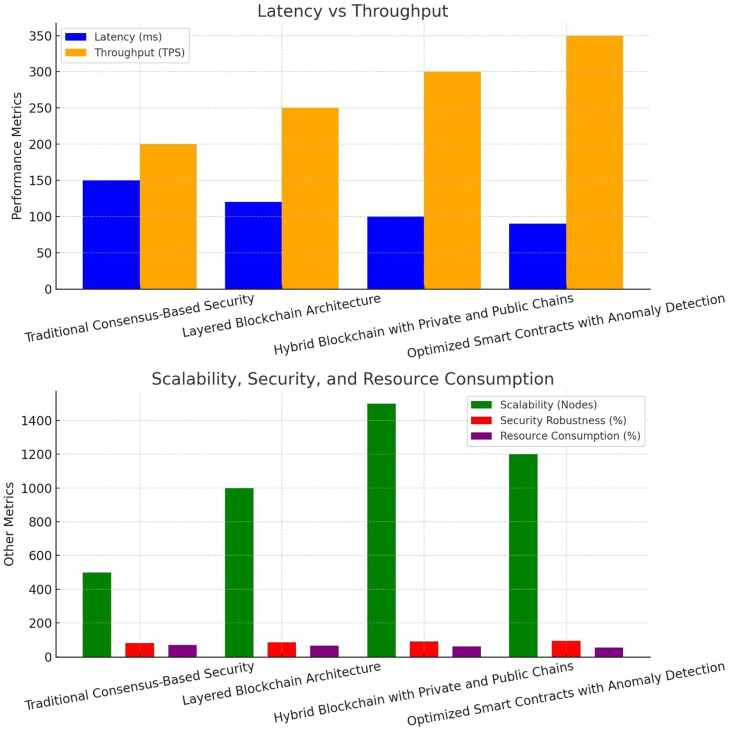
Comparative analysis of exiting approach.

Conclusively, the Optimized Smart Contracts with Anomaly Detection approach results in providing the most balanced SDVN security framework in terms of efficiency, security, and robustness. The other approaches are also highly useful especially if only certain parameters are needed to be focused on. The comparative analysis of these approaches has been presented in the following Tables 1 and 2, Fig. 2.

**Table 1:** Research gap.

Author	Method	Advantage	Limitation	Research Gap
Zhang and Li [2]	Layered blockchain	Improved scalability	High architectural complexity	No adaptive consensus
Nguyen and Tran [4]	Latency-aware consensus	Reduced latency	Not scalable in large networks	No dynamic protocol selection
Kim and Park [5]	Blockchain access control	Strong authentication	Performance degradation	Lack of anomaly detection
Liu and Zhou [10]	ML anomaly detection	Early threat detection	High computational cost	No blockchain integration

**Table 2:** Comparative analysis using different approaches.

Parameter	Traditional Consensus-Based Security	Layered Blockchain Architecture	Hybrid Blockchain with Private and Public Chains	Optimized Smart Contracts with Anomaly Detection
Latency (ms)	150	120	100	90
Throughput (TPS)	200	250	300	350
Scalability (Nodes)	500	1000	1500	1200
Security Robustness	80 %	85 %	90 %	95 %
Resource Consumption	70 %	65 %	60 %	55 %

**Fig. 3:**
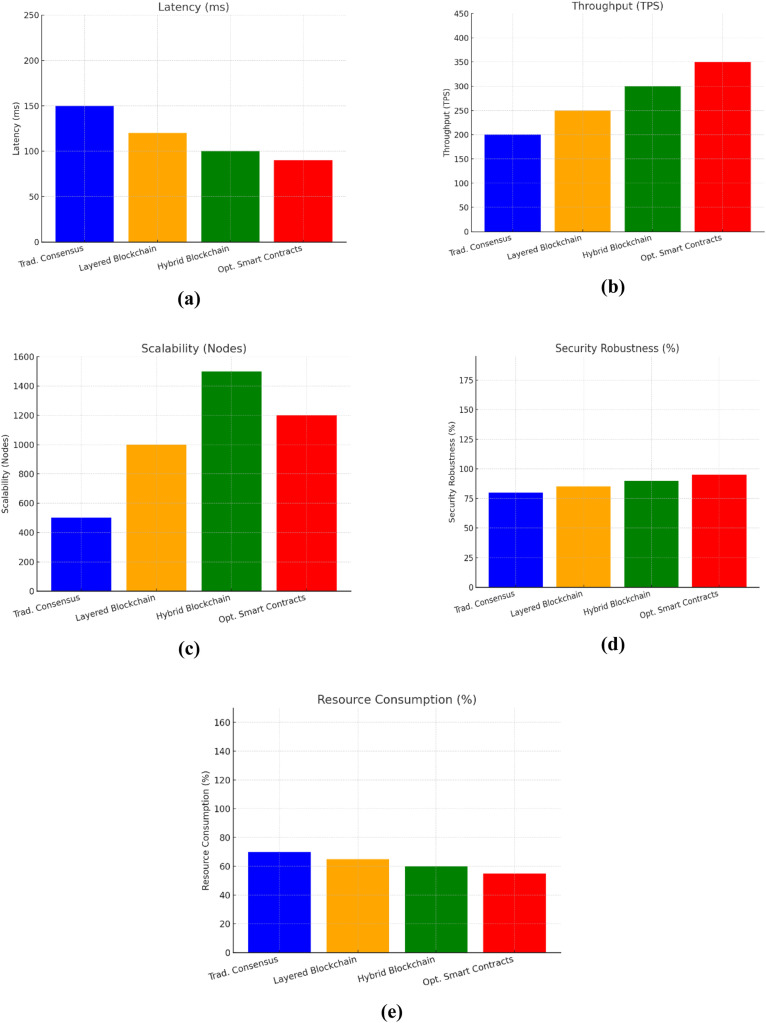
Analysis using different parameters a) Latency (ms) b) Throughput (TPS) c) Scalability (Nodes) d) Security Robustness ( %) e) Resource Consumption ( %).

Fig. 3 also presents a comparison between performance of the above-mentioned approaches in the context five key metrics, including latency, throughput, and security robustness, scalability, and resource effectiveness. The latency figure and throughout graphics present Optimized Smart Contracts with Anomaly Detection as the most suitable approach for time-sensitive tasks and effective management of huge data respectively. It has the lowest latency and fastest transaction speed.

The Hybrid Blockchain with Private and Public Chains approach, having the best scalability score, emerges as the most suitable process for large-scale blockchain applications. Moreover, the Optimized Smart Contracts with Anomaly Detection approach seems to be the most suitable one, based on its highest security robustness score, for ensuring SDVN integrity. It provides the most advanced protection to the SDVNs against the potential cyberattacks.

In terms of efficient consumption of resources, the Optimized Smart Contracts with Anomaly Detection approach showcases the best outcomes, with the score of 55 % resource consumption. It utilizes the least amount of CPU as compared to other three approaches. The Resource Consumption figure illustrates the comparison in this regard in an effective way. To sum the results up, this strategy gains the best outcomes as compared to other approaches, which makes it the most effective solution to ensure SDVN security and integrity? However, each strategy has its own strengths which can be utilized as per requirements of a particular network.

In the advancing automobiles industry, the blockchain-backed approach proposed in this study is widely applicable in terms of securing SDVNs. For example, this methodology can be applied to fulfill secure and integral vehicle-to-vehicle communication requirements. Ultimately, it can help in boosting the potential of advanced driving assistance systems (ADAS) and making various vehicle operations safe and secure. The huge amount of data generated by these systems does not limit the applicability of this framework as it showcases higher scalability, reduced latency, and increased throughput simultaneously.

This work introduced blockchain-based security system of Software-Defined Vehicular Networks (SDVNs) to overcome the emerging needs of secure and reliable vehicular communication. The framework proposed is a multi-layer blockchain architecture with smart security solutions to improve the integrity of data, scalability, and the reliability of the network. Adaptive Consensus Selection Algorithm (ACSA) was proposed in order to choose the most appropriate consensus protocol dynamically, depending on the real time network characteristics like node density, rate of transacting and latency. Moreover, to decrease the cost of smart contracts implementation without losing the necessary level of network security, a mathematical optimization model was designed. An anomaly detection module, which is a machine learning-related module, was implemented at the edge layer to identify malicious nodes and abnormal vehicle behavior in real time. The experimental analysis with the help of the vehicle network simulation surroundings proved that the proposed framework increases the system performance in the aspects of reduction of the latency, increase of the throughput, and the security robustness in the cases of the traditional blockchain-based methodology. It is shown that the interaction of adaptive blockchain mechanisms with intelligent edge-based detection can contribute greatly to the security as well as efficiency of the next-generation SDVN systems.

## Limitations

None.

## Ethics statements

None.

## Credit author statement

Sonali Patil 1 conceived and authored or reviewed drafts of the paper, and approved the final draft.

Deepali Naik 2 conceived and designed the experiments, performed the experiments, analyzed the data, performed the computation work, prepared figures authored or reviewed drafts of the paper, and approved the final draft.

Madhura Kalbhor3 conceived and designed the experiments, performed the experiments, analyzed the data, performed the computation work, prepared figures.

Aparna Joshi4 conceived and designed the experiments, prepared figures authored or reviewed drafts of the paper, and approved the final draft

## Funding

The authors received no funding for this work.

## Declaration of competing interest

The authors declare that they have no known competing financial interests or personal relationships that could have appeared to influence the work reported in this paper.

## Data Availability

Data will be made available on request.
